# Videoendoscopic gracilis muscle flap (VEGMF): A new minimally invasive technique for rectovesical fistula closure

**DOI:** 10.1590/S1677-5538.IBJU.2019.0626

**Published:** 2020-09-02

**Authors:** Marcos Tobias-Machado, William Enrique Pertuz Genes, Anis Taha, Fernando Cunha Lorenzetti, Felipe Quadros, Hamilton Campos Zampolli, Liseth viviana Echavez Pacheco

**Affiliations:** 1 Departamento de Urologia Faculdade de Medicina do ABC Santo AndréSP Brasil Departamento de Urologia, Faculdade de Medicina do ABC, Santo André, SP, Brasil;; 2 Divisão de Urologia Instituto do Câncer Dr. Arnaldo Veira de Carvalho São PauloSP Brasil Divisão de Urologia Instituto do Câncer Dr. Arnaldo Veira de Carvalho, São Paulo, SP, Brasil;; 3 Corporación Universitaria Rafael Nuñez Cartagena Colômbia Corporación Universitaria Rafael Nuñez, Cartagena, Colômbia

## Abstract

Fistulas between the rectum and the bladder have different etiologies, they may be congenital, acquired, or iatrogenic. Symptoms such as pneumaturia, fecaluria, and the passage of urine through the rectum are often alleviated by fecal and urinary diversion. The interposition of a flap of gracilis muscle is a technique that is very successful, but the conventional dissection of this flap involves a very large incision and a worse aesthetic result *.*

## INTRODUCTION

Prostate cancer is one of the most common malignancies that endanger geriatric males. Its global incidence rate ranks second among those of all male malignancies. In 2012, there were approximately 1.1 million new cases of prostate cancer in the World (1). The occult onset of prostate cancer has no or unapparent symptoms in the early stage. Besides, tumor metastasis remains the main obstacle for prostate cancer treatment and the main cause of death (2).

## OBJECTIVES

To present a new minimally invasive technique for the dissection and transposition of a gracilis muscle flap (VEGMF) for the correction of rectovesical, rectovaginal and rectourethral fistulas.

## MATERIALS AND METHODS

Case report: A 75 years old male, with rectovesical fistula, with a history of laparoscopic radical prostatectomy in May 2018, with perforation of the rectum and immediate closure in 2 planes, with subsequent fistula development, with subsequent colostomy. The patient was submitted to conventional perineal fistula closure and (VEGMF) as a graft between reconstructed planes to prevent recurrence.

### Surgical technique (VEGMF)

1. External rotation of abducted limb
2. Identification by palpation of gracilis muscle and insertion tendon
3. 2.5cm incision above of gracilis insertion tendon
4. External dissection and tendon repair
5. Single port placement
6. Optical blunt dissection between skin and gracilis muscle
7. Co2 Insuflation (10-15mmHg)
8. Dissection of gracilis muscle with ultrasonic energy under optical vision as far as possible
9. Removal of single port and external cutting of gracilis tendon
10. Single port replacement and insuflation
11. Grasped divided gracilis tendon is carried near to perineal incision
12. Kelly forceps passed under the skin through perineal incision to grasped gracilis
13. Exteriorization of gracilis muscle flap by the perineal wound without any tension, preserving the major vascular pedicle
14. Interposition between bladder and rectum
15. Muscle flap fixation

## RESULTS

Surgical time: 240 min, without clinical complications, 3 days of hospital stay, urethrovesical catheter removal at day 30, with cystoscopy, checking the closure of the fistula, reconstruction of the gastrointestinal tract without complications or fistula recurrence.

## CONCLUSIONS

This technique is a feasible and effective method for the treatment of rectourethral fistula, providing a viable tissue flap between the rectum and the bladder. This procedure seems to be associated with low morbidity and best cosmetics results in comparison with conventional technique.

